# Electrical impedance tomography to aid in the identification of hypoxemia etiology: Massive atelectasis or pneumothorax? A case report

**DOI:** 10.3389/fmed.2022.970087

**Published:** 2022-09-02

**Authors:** Runshi Zhou, Chaokai He, Yi Chi, Siyi Yuan, Bo Tang, Zunzhu Li, Qi Li, Huaiwu He, Yun Long

**Affiliations:** Department of Critical Care Medicine, Peking Union Medical College Hospital, Peking Union Medical College, Chinese Academy of Medical Sciences, Beijing, China

**Keywords:** EIT, lung perfusion and ventilation, massive atelectasis or pneumothorax, case report, electrical impedance tomography

## Abstract

**Background:**

Bedside ultrasound is often used to determine the etiology of hypoxaemia, but not always with definitive results. This case reports the application of electrical impedance tomography (EIT) and saline injection to determine the etiology of hypoxaemia in a complex case that could not be identified by bedside ultrasound. The determination of the etiology of hypoxaemia by EIT and saline injection, regional ventilation and perfusion information can be used as a new clinical diagnostic method.

**Case presentation:**

A post-cardiac surgery patient under prolonged mechanical ventilation for lung emphysema developed sudden hypoxemia in the intensive care unit (ICU). A line pattern and lung sliding sign abolishment were found in the left lung, but there was no evidence of a lung point sign on bedside ultrasound. Hence, the initial diagnosis was considered to be a massive pneumothorax. To further define the etiology, EIT and saline bolus were used to assess regional ventilation and perfusion. A massive ventilation defect was found in the left lung, in which regional perfusion was maintained, resulting in an intrapulmonary shunt in the left lung. Finally, the conjecture of a pneumothorax was ruled out considering the massive atelectasis. After the diagnosis was clarified, hypoxaemia was corrected by restorative ventilation of the left lung after changing the patient's posture and enhancing sputum drainage with chest physiotherapy.

**Conclusions:**

This was the clinical case involving EIT and saline bolus to establish the differential diagnosis and guide clinical decisions for patients with acute hypoxemia. This study highlighted that combination regional ventilation, EIT perfusion, and saline bolus provided helpful information for determining the etiology of hypoxemia. The results of this study contribute to the development of emergency patient management.

## Background

Interpreting ventilation-perfusion matching is essential in the differential diagnosis of acute hypoxemia. Electrical impedance tomography (EIT) is a new, non-invasive, radiation-free, bedside lung imaging method, that has gained attention in the diagnosis of acute respiratory failure (ARF), such as pleural effusion and pneumothorax (1, 2). Moreover, the saline bolus-based EIT method has been validated against electron beam computed tomography (CT) imaging for assessing regional lung perfusions (3). Hence, combination regional ventilation with perfusion data derived from EIT and saline bolus significantly aids in the bedside diagnosis of ARF etiology. We report a case involving the application of EIT and saline bolus to determine the etiology of an acute hypoxemia patient with a suspected pneumothorax on bedside ultrasound. This case had been enrolled in a previous study (4).

## Case presentation

**Figure 1:**
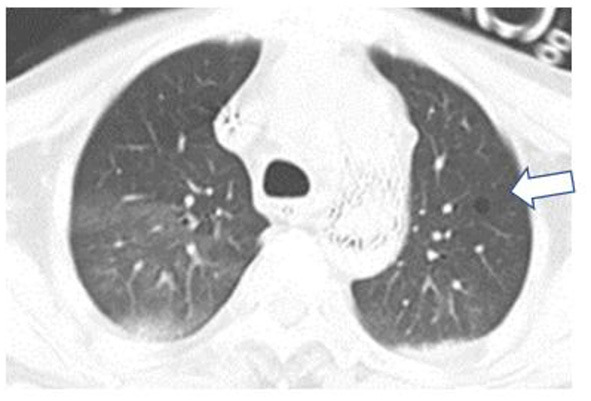
The computed tomography (CT) scans of the patient (baseline). A baseline lung CT scan noted emphysema and pulmonary bulla in the left lung. The arrow in this figure points to the pulmonary bulla of lung.

**Figure 2:**
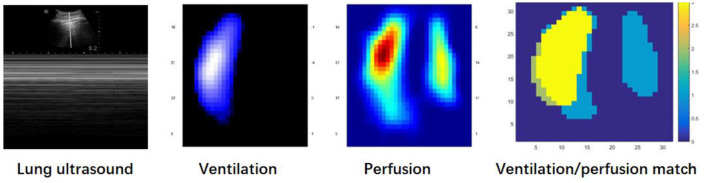
The lung ultrasound and EIT monitoring (hypoxemia occurs). 1. Lung ultrasound imaging with a convex probe (5 MHz) showed the absence of gliding sign, confirmed by the Barcode sign in M-mode at the onset of hypoxemia. 2. Functional electrical impedance tomography (EIT) images of ventilation and perfusion distribution at the onset of hypoxemia. Low-ventilated regions are marked in dark blue and high-ventilated regions in white. Regions with high perfusion are marked in red and low perfusion in blue. A massive ventilation defect was found in the left lung in which perfusion was maintained, and perfusion and ventilation matched the image at the onset of hypoxemia. Regions with high ventilation and low perfusion (indicate dead space) are marked in light green; low ventilation and high perfusion regions (indicate intrapulmonary shunt) in light blue; good ventilation-perfusion matching in yellow.

**Figure 3:**
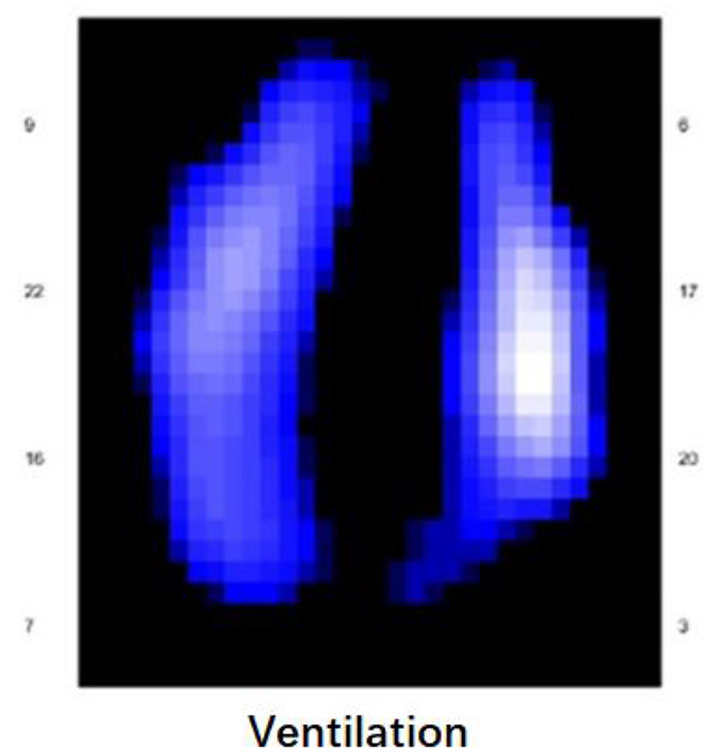
The results of the EIT assessment of the patient's lungs (post-treatment).

**Figure 4:**
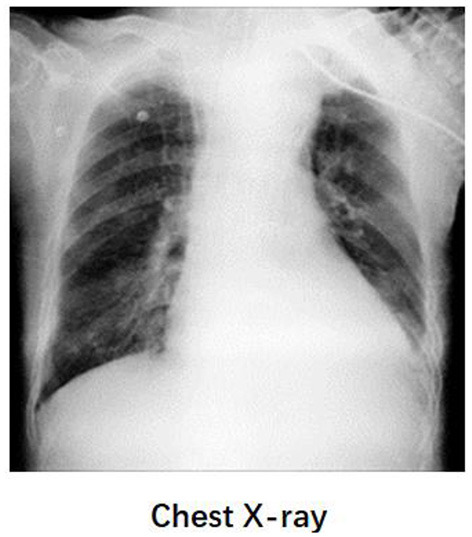
The chest X-ray of patient. Chest radiography did not show signs of pneumothorax. Functional EIT images of ventilation showed that the defect of the left lung had been restored after treatment.

A 58-year-old post-cardiac surgery patient, who underwent prolonged mechanical ventilation [pressure support (PS) mode: PS 12 cmH_2_O, positive end-expiratory pressure 5 cmH_2_O, fraction of inspired oxygen 40%] for lung emphysema, developed sudden dyspnea and severe hypoxemia [peripheral oxygen saturation (SpO_2_) decreased from 99 to 76%] in the ICU. The patient had a heart rate of 110-120 bp/min and blood pressure of 120-130/75-90 mmHg. A previous CT scan showed emphysema and a pulmonary bulla in the left lung (Figure 1). The medical team immediately performed an emergency bedside ultrasound on the patient. A line pattern and lung sliding sign abolishment were found in the left lung (Figure 2), but there was no evidence of a lung point sign on emergency bedside ultrasound. A massive pneumothorax was suspected. Immediately afterwards, the doctor called the radiology department for a bedside chest X-ray scan. In the meantime, the EIT and saline bolus were used to assess the regional ventilation and perfusion. A massive ventilation defect was found in the left lung, which had normal perfusion (Figure 2). This suggested an intrapulmonary shunt in the left lung. Massive atelectasis, rather than pneumothorax, was considered. After enhancing sputum drainage by changing the patient's posture and chest physical therapy, the SpO_2_ improved, and the left lung ventilation recovered (Figure 3). The radiology staff arrived at the ICU unit to perform the scan later than the EIT assessment was completed. The bedside chest X-ray excluded pneumothorax (Figure 4). When the patient was successfully weaned from the ventilator, he was transferred to the regular ward 19 days after ICU admission.

## EIT methods

Electrical impedance tomography measurements were performed using PulmoVista (Dräger Medical, Lübeck, Germany). During the protocol, a silicone EIT belt with 16 surface electrodes was placed around the patient's thorax in one transverse plane corresponding to the fourth intercostal parasternal space, and then connected to the EIT monitor. A bolus of 10 ml 10% NaCl was injected through the central venous catheter during an 8s respiratory pause. The ventilation maps were derived from a previous publication, where the impedance changes reliably tracked local changes in the air content within the lung, pixel by pixel (1). The perfusion maps were obtained by injecting a bolus of 10 ml hypertonic solution (NaCl 10%) through a central venous catheter during an expiratory breath-hold for at least 8 s. Due to its high conductivity, NaCl 10% acted as an EIT contrast agent, which passed through the pulmonary circulation after being injected into the right atrium, thereby producing a dilution curve following the typical first-pass kinetics. The resulting regional time impedance curves were then analyzed to quantitatively assess regional perfusion (3, 5).

## Discussion and conclusions

This case demonstrated the application of combined ventilation with perfusion data from EIT and saline bolus to establish the differential diagnosis of acute hypoxemia. EIT has been used in several animal studies and clinical cases to identify pneumothorax by regional ventilation defection (6, 7).

However, regional ventilation defection results from various intrathoracic pathologies, such as atelectasis, hemothorax, and pneumothorax. Without additional imaging data, it is difficult to establish the differential diagnoses clinically. EIT has been used as a diagnostic tool for atelectasis. EIT is currently used in clinical practice to monitor the patient's lung ventilation, which can indirectly assist the clinician in determining the patient's lung condition. In this case the patient was in a unique situation and we administered saline as soon as the patient's condition allowed, which helped us to further determine the patient's condition and identify the patient as having pulmonary asplenia. The combined regional ventilation and perfusion enhances EIT performance in determining lung pathology. Tingay et al. reported the use of EIT to measure perfusion and ventilation patterns in a newborn with impaired perfusion and ventilation in a single lung (8).

In the present case, we auscultated the patient's lungs and found a reduced breath sound, but no positive signs on percussion. We immediately performed a bedside lung ultrasound and EIT. A massive pneumothorax was suspected based on a “regional ventilation defection,” and “A line pattern and lung sliding sign abolishment” on bedside EIT and ultrasound examination. However, pneumothorax could not be determined without findings of the lung point sign. The presence of the lung sliding sign excluded pneumothorax, but the absence of lung sliding sign can be related to other conditions, such as phrenic nerve paralysis, pleural adhesions, pulmonary contusion, emphysema, and obesity (8). Moreover, the patient had emphysema in the related lung region. Anna Del Colle et al. reported that the lack of the “gliding sign” mimicked pneumothorax in patients with severe asthmatic patients (9). Boccatonda et al. found that lung sliding sign was abolished by lung atelectasis, but not pneumothorax (10). That was due to adhesions caused by solid lung lesions and the lung sliding sign may disappear.

The present case supported the utility of the combination of regional ventilation and perfusion, assessed by saline injection, in differentiating between pneumothorax and atelectasis, and making an accurate clinical decision. The lung regional perfusion and saline bolus helped identify massive intrapulmonary and extrapulmonary lesions. A similar change in regional ventilation defection with normal perfusion was observed in the one-lung ventilation animal model with capnothorax (5). Moreover, several animal studies have found that the indicator-based EIT method determined the regional lung perfusion defect after a pulmonary embolism-like event (3).

This patient was enrolled in a clinical study of using lung perfusion and ventilation by EIT for respiratory failure etiology. The related examinations for massive atelectasis or pneumothorax were performed based on clinical regulations, and the EIT examination did not affect the clinical therapy in this case. Because the lung point sign was not observed, the bedside ultrasound could not make a definitive diagnose of pneumothorax. Moreover, the chest X-ray examination usually takes about 30-60min since there was no mobile X-ray machine at the bedside in our department. Hence, the EIT examination was a reasonable option, which has potential advantages for early diagnoses. We found that the EIT examination was helpful for the differential diagnoses of massive atelectasis or pneumothorax in this case. The diagnosis of EIT corresponded with the result of the following Chest-X ray examination. The patient's atelectasis improved after physiotherapy of the lungs and therefore no definite atelectasis images were found on chest X-ray. This case suggested that saline bolus EIT examination might be an early and alternative method for respiratory failure etiology during the waiting period for Chest-ray under some difficult conditions. Caio et al. found that EIT Monitoring could early identify pneumothorax appearance during recruitment maneuvers in ARDS patients (11). Moreover, the influence of the 10 mL 10% NaCl on hydro electrolyte balance was minimal. Several clinical studies have validated the safety of the method (12).

At last, we acknowledge that monitoring and judgment of pulmonary circulation using EIT is still not well established. The case, as a proof concept, will significantly contribute to the development of emergency patient management by saline bolus EIT examination.

## Data availability statement

The original contributions presented in the study are included in the article/supplementary material, further inquiries can be directed to the corresponding authors.

## Ethics statement

This study was approved by the institutional review board of PUMCH (approval number: JS-1896). The patients/participants' family provided written informed consent to participate in this study.

## Author contributions

YL and HH conceived the original idea. RZ carried out the research and wrote the manuscript with support from HH, CH, and YC. QL verified the numerical results of RZ. SY, BT, and ZL supervised the findings of this work and provided support in writing the manuscript. All authors have discussed the results and contributed to the final manuscript.

## Funding

This work was funded by the Non-profit Central Research Institute Fund of Capital's Funds for Health Improvement and Research (No. 2020-2-40111), Excellence Program of Key Clinical Specialty of critical care medicine of Beijing in 2020, Beijing Municipal Science and Technology Commission (No. Z201100005520051).

## Conflict of interest

The authors declare that the research was conducted in the absence of any commercial or financial relationships that could be construed as a potential conflict of interest.

## Publisher's note

All claims expressed in this article are solely those of the authors and do not necessarily represent those of their affiliated organizations, or those of the publisher, the editors and the reviewers. Any product that may be evaluated in this article, or claim that may be made by its manufacturer, is not guaranteed or endorsed by the publisher.

## Abbreviations

ARF: acute respiratory failure
CT: computerized tomography
EIT: electrical impedance tomography
ICU: intensive care unit
PS: pressure support
SPO_2_: peripheral oxygen saturation.
